# Antibacterial and Biocompatible Polyethylene Composites with Hybrid Clay Nanofillers

**DOI:** 10.3390/ma16145179

**Published:** 2023-07-23

**Authors:** Lenka Klecandová, Damian S. Nakonieczny, Magda Reli, Gražyna Simha Martynková

**Affiliations:** 1 IT4Innovations, VSB–Technical University of Ostrava, 17 Listopadu 2172/15, 708 00 Ostrava-Poruba, Czech Republic; lenka.pazourkova@vsb.cz; 2 Department of Biomedical Engineering, Silesian University of Technology, Akademicka 2A, Młyńska 8, 44-100 Gliwice, Poland; damian.nakonieczny@polsl.pl; 3 Intitute of Environmental Technologies, CEET, VŠB—Technical University of Ostrava, 17 Listopadu 2172/15, 708 00 Ostrava-Poruba, Czech Republic; magda.reli@vsb.cz; 4 Nanotechnology Centre, CEET, VSB—Technical University of Ostrava, 17 Listopadu 2172/15, 708 00 Ostrava-Poruba, Czech Republic

**Keywords:** polyethylene, nanoclays, hydroxyapatite, chlorhexidine diacetate, antibacterial, biocompatible, composites

## Abstract

Low-density polyethylene is one of the basic polymers used in medicine for a variety of purposes; so, the relevant improvements in functional properties are discussed here, making it safer to use as devices or implants during surgery or injury. The objective of the laboratory-prepared material was to study the antimicrobial and biocompatible properties of low-density polyethylene composites with 3 wt. % hybrid nanoclay filler. We found that the antimicrobial activity was mainly related to the filler, i.e., the hybrid type, where inorganic clay minerals, vermiculite or montmorillonite, were intercalated with organic chlorhexidine diacetate and subsequently decorated with Ca-deficient hydroxyapatite. After fusion of the hybrid nanofiller with polyethylene, intense exfoliation of the clay layers occurred. This phenomenon was confirmed by the analysis of the X-ray diffraction patterns of the composite, where the original basal peak of the clays decreased or completely disappeared, and the optimal distribution of the filler was observed using the transmission mode of light microscopy. Functional property testing showed that the composites have good antibacterial activity against *Staphylococcus aureus*, and the biocompatibility prediction demonstrated the formation of Ca- and P-containing particles through an in vitro experiment, thus applicable for medical use.

## 1. Introduction

Research on hybrid inorganic–organic nanocomposites has intensified in recent decades. Nanocomposites of polymers with pure or modified clay minerals are of particular interest. Minerals from the smectite group are more often used as nanofillers in polymer composites than minerals from the vermiculite group. In general, the addition of nanoclays to a polymer can be a way to improve many of the polymer’s shortcomings, such as the mechanical properties of stiffness, tensile strength, and toughness or the barrier properties of permeability to gases and moisture, which is useful in applications such as food packaging. A similar positive effect is observed in improved fire resistance or an increase in the thermal stability of the polymer, which allows it to withstand higher temperatures. These properties may vary depending on the type and amount of clay used, as well as the processing conditions of the nanocomposite [1].

Polymer composites are widely used in medicine, for example, for bone tissue engineering [2], as surgical devices (miniscrews) [3], biomaterials [4], implant coatings [5], etc. Implanted devices are often associated with bacterial infection, which poses a significant threat to patients. High infection rates (2–6%) are observed in orthopedic implants, dental appliances, vascular grafts, urinary catheters, and venous catheters [6,7,8,9]. The development of a polymer composite with antibacterial and also biocompatible properties is essential.

Polyethylene (PE) is a widely used polyolefin with a simple molecular structure written by the formula (CH_2_)n. PE is used to replace or augment damaged bone or cartilage in the body. It is commonly used in orthopedic surgeries such as joint replacements, spinal fusions, and reconstructive surgeries [10]. Polyethylene is recognized as high-density (HDPE) and low-density polyethylene (LDPE). The LDPE contains many paraffinic branches and has zig zag storage of carbon in the chains [11]. Polyethylene is a durable and biocompatible material that can withstand the stresses and pressures of daily use in the body. Polyethylene implants, which are generally safe and effective, can sometimes cause complications, such as infection, implant loosening or wear, and inflammation. However, it is important to note that the biocompatibility of any material depends on a variety of factors, including the specific grade of the material, its processing methods, and its intended use. While PE is generally considered biocompatible, specific modifications or additives can affect its biocompatibility. Therefore, it is essential to consult the relevant material specifications and regulatory guidelines when considering the use of PE in medical applications. The incorporation of organic or inorganic substances with antibacterial properties can improve the antibacterial behavior of the entire implant. Clay minerals are widely used natural layered materials for improving polymers’ weak points such as their low mechanical or thermal stability. The major advantage of clays is the low cost of the abundant mineral, the ease of processing, and their nontoxicity for wide range of biological systems. There are several studies targeting the preparation of antimicrobial polymer composites using modified clay minerals [12,13,14]. The montmorillonite and vermiculites are the most commonly used 2:1 layered phyllosilicates in many industrial applications. They are built of an aluminosilicate nanometric layer based on two tetrahedral sheets and one octahedral sheet in between them, and the characteristic charge on the layer is caused by the disbalance of the isomorphous substitution of Si/Al. Such charge allows holding in the interlayer space the original or exchanged inorganic cation or optionally exchanging the inorganic cation for an organic one. The vermiculites and smectites are a suitable substrate for antimicrobial activity. The antimicrobial species attaches on the clay mineral surface and is gradually released, which ensures a long-term antimicrobial effect [15,16].

For application in medicine, another important factor is the biocompatibility of the prepared nanomaterials. Materials with good biocompatibility are supportive for the growth of osteoblasts cells [17]. Hydroxyapatite is a mineral form of calcium apatite that closely resembles the composition of natural bone. It is a key component of the inorganic matrix of bone and teeth. When used in the context of bone regeneration and orthopedic applications, hydroxyapatite demonstrates osteoconductive properties. Human bone is a Ca-deficient hydroxyapatite (CDH) with the chemical formula Ca_10−*x*_(HPO_4_)*_x_*(PO_4_)_6−*x*_(OH)_2−*x*_ (0 < *x* < 1). CDH can serve for non-biological applications as a catalyst, a component for mordants, an inert filler in drug pellets, a precursor for the preparation of organic phosphates, etc., [18,19].

Chlorhexidine is an antiseptic and disinfectant commonly used in healthcare settings to reduce the risk of infection. It is a broad-spectrum antimicrobial agent effective against a wide range of bacteria, fungi, and some viruses [20,21].

Hydroxyapatite, clay mineral, and polymer composites have been studied for their processability, their improvement of polymer properties, or to obtain additional functional properties. Utilization as biomaterials in tissue engineering [22,23,24,25] has become a good alternative to the conventional polymeric one. Katti et al. [2] studied a nanocomposite of chitosan and montmorillonite/hydroxyapatite. The composite exhibited better properties than composites composed only from MMT/chitosan or HAp/chitosan [2]. Ambre et al. studied a composite of in situ hydroxyapatite prepared in an interlayer space of organically modified clay mineral. This hybrid clay was used as filler for a polymer composite. The composite showed biocompatibility and played an important role in the process of bone nodule formation [4]. Scalable fabrication technologies that enable control over architecture at multiple scales, including three-dimensional printing and electric-field-assisted techniques, can then be employed to process these biomaterials into suitable forms for bone-tissue engineering [26].

Our study was focused on the laboratory preparation of a polymer nanocomposite with an antibacterial and biocompatible clay hybrid nanofiller. The aim of the work was to obtain the desired functional properties by combining polyethylene with vermiculite or montmorillonite filler, which was intercalated with chlorhexidine diacetate and then modified with Ca-deficient hydroxyapatite. The structures of the final polymer nanocomposites were studied using X-ray diffraction (XRD) analysis, infrared spectroscopy (FTIR), and differential scanning calorimetry (DSC). The particle size distribution of the clay nanofillers was measured and observed using light microscopy and laser scattering methods. Finally, the antibacterial activity against Gram-positive *Staphylococcus aureus* was studied. Biocompatibility tests, according to Kokubo [27], were performed by soaking polymer nanocomposites in simulated body fluids (SBF).

## 2. Materials and Methods

### 2.1. Materials and Synthesis

The synthesis of the composite consisted of three steps: intercalation–decoration–compounding.

Chlorhexidine diacetate (C_22_H_30_Cl_2_N_10_, M = 505.45 g·mol^−1^) purchased from Sigma Aldrich (Czech Republic) was used for the intercalation of clays. Sodium phosphate dibasic dodecahydrate (Na_2_HPO_4_∙12 H_2_O, M = 358.14 g·mol^−1^) and calcium chloride dihydrate (CaCl_2_∙2 H_2_O, M = 147.01 g·mol^−1^) purchased from Vitrum VWR, Co. (Stříbrná Skalice, Czech Republic) were used as the CDH precursors.

The polyethylene powder and pellet mixture were made from the industrial Low-Density PE (LDPE) Bralen VA 20-60 (Slovnaft, Bratislava, Slovak Republic). 

The composite of clay mineral/CA/CDH was prepared according to our previous study [19]. Firstly, vermiculite (V) or montmorillonite (M) were intercalated with chlorhexidine diacetate (CA) using a cation exchange process in a wet process (VCA or MCA, respectively). The next step was the decoration of the intercalated clays with CDH particles. The CaCl_2_ solution was slowly added to a solution of Na_2_HPO_4_, which contained VCA or MCA. The precipitate was allowed to sediment for 24 h. After this period, the supernatant was decanted, and the precipitate was dried at 70 °C for 12 h. The samples were marked as MCAH (for MCA + CDH) and VCAH (for VCA + CDH).

Polyethylene nanocomposites were prepared from the mixtures containing 38.8 g of LDPE mixture and 1.2 g (3 wt. %) of nanofillers MCAH and VCAH, respectively. Each mixture was blended in the Brabender single screw extruder 19/25 kneading chamber (Brabender GmbH & Co., Duisburg, Germany) at 160 °C for 10 min in two rate intervals (10 rpm for 2 min and subsequently 50 rpm for 8 min). Then, the matter was pressed at 160 °C into the 1 mm thick plates of size 100 mm × 100 mm. The polyethylene (PE) composites were marked as VCAH/PE and MCAH/PE.

### 2.2. Methods for Characterization

The composites were studied employing X-ray powder diffraction (XRD) methods using an X-ray diffractometer Rigaku Ultima IV (Tokyo, Japan) (reflection mode, Bragg-Brentano arrangement, CuKα_1_ radiation, working condition 40 kV, 40 mA) in the ambient atmosphere under constant conditions. The phase analysis of the studied samples was performed based on the Database JCPDS ICDD PDF4+(2022).

The images of samples were performed by scanning electron microscopy (SEM) on a PHILIPS XL-30 (FEI, Hillsboro, OR, USA) equipped with an energy dispersive spectrometer (EDS). The samples were coated with gold/palladium to ensure the good conductivity for surface examination, and a secondary electrons detector was used.

The differential scanning calorimetry (DSC) using Setaram DSC 131 evo (Caluire, France) calorimeter (from −25 to 150 °C, 5°/min, Ar atmosphere) was used to study the thermal properties of the composites.

The images of the composite were acquired using light microscopy (LM) on an Olympus BX51 (Tokyo, Japan) equipped with a camera Olympus UC30, using bright field polarized light at transmission mode.

The particle size of the clay hybrid filler was measured using sizer HORIBA Partica LA-950 (Tokyo, Japan) in distilled water (used refractive indexes for water 1.33, vermiculite 1.54, montmorillonite 1.49).

### 2.3. Antibacterial Tests

The antibacterial activity was studied against Gram-positive *Staphylococcus aureus*.

After 24 h, 48, 72, and 96 h incubation under similar conditions, the active bacteria were counted.

An antibacterial test of the prepared LDPE nanocomposites was performed by the microbial fingerprints technique. The method assumes that the bacteria under the same conditions gradually die. Each sample plate was cut to the three squared plates (25 cm^2^). Then, 100 μL of the bacterial suspension of Gram-positive *S. aureus* CCM 3953 (1.0 × 10^6^ cfu.mL^−1^), provided by the Czech collection of microorganisms (CCM), was spread on the plates and was left to dry in the laminar box at 21 °C. Then, the dried bacterial suspension on the surface of plates was stamped using the microbial fingerprints technique on the three discs with blood agar in 24, 48, 72 and 96 h time intervals. The bacteria cultivation took place in the thermostat at 35 °C for 24 h. The number of colony-forming units of bacteria (CFU) at all three fingerprints were counted and averaged.

### 2.4. In Vitro Biocompatibility

The in vitro biocompatibility test was carried out according to Kokubo et al. [27] by soaking the samples in simulated body fluid (SBF) solution.

The required amount of SBF solution for each sample is calculated as follows:

V_s_ = S_a_/10,

where V_s_ is the volume of the SBF (mL), and S_a_ is the apparent surface area of the specimen (mm^2^).

The calculated volume of the SBF was put into a plastic bottle. After heating the SBF to 36.5 °C, a specimen was placed in the SBF so that the sample was hanging in the bulk of the testing container, not touching the walls of the container. The solution was changed every week for simulation of the dynamic conditions in the human body, and after soaking for a period of 4 weeks in the SBF, the specimens were removed from the SBF and gently washed with pure water. Then, the samples were dried at laboratory temperature and placed in a desiccator [27].

The formation of the layer on the surface of the immersed samples was investigated using a scanning electron microscope.

## 3. Results and Discussion

For all stages of sample preparation, the characterization of the properties and parameters was carried out.

### 3.1. X-ray Diffraction Phase Analysis

**Figure 1:**
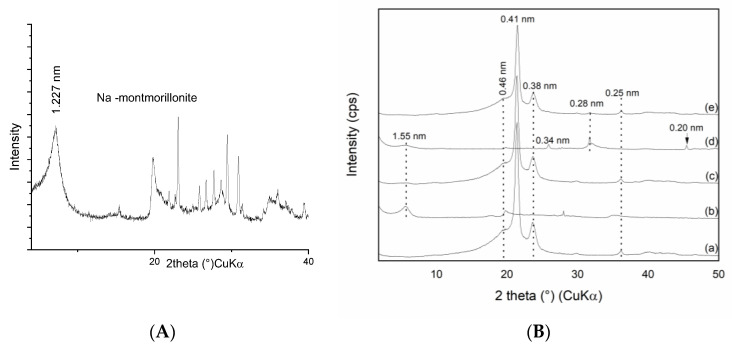
XRD patterns of the samples of (**A**) monoionic Na-montmorillonite and (**B**) (a) pure PE, (b) MCA, (c) PE/MCA, (d) MCAH, and (e) PE/MCAH.

Figure 1A shows the monoionic Na-montmorillonite XRD pattern and Figure 1B(a) the patterns of pure polyethylene and as well as the fillers MCA and MCAH followed by the complete PE composites. The pure PE pattern presented mainly two intensive reflections at d_(001)_ = 0.41 nm and d_(200)_ = 0.38 nm, which corresponded to the orthorhombic structure of PE [28,29]. The PE pattern contained another weak reflection d = 0.46 nm evaluated as PDF card no. 00-011-0834. The filler MCA showed a diffused basal reflection of montmorillonite intercalated with chlorhexidine diacetate at d = 1.55 nm [12] corresponding to the lateral alignment of large CA molecules (Figure 1B(b)). After decoration with CDH, the MCAH pattern showed in both phases a basal reflection of intercalated montmorillonite at d = 1.55 nm and basal reflections of CDH at d = 0.334 and 0.28 nm, as shown in Figure 1B(d). The diffraction patterns of the PE composites showed minimal peak traces of intercalated MCA and regular peaks of PE in PE/MCA and PE/MCAH, as shown in Figure 1B(c,e), respectively. Based on this observation, we can estimate that montmorillonite exfoliation took place in both composites.

The polyethylene composite with MCAH filler exhibited intensive basal reflections of PE and a reflection of CDH at d = 0.28 nm (Figure 1B(e)).

**Figure 2:**
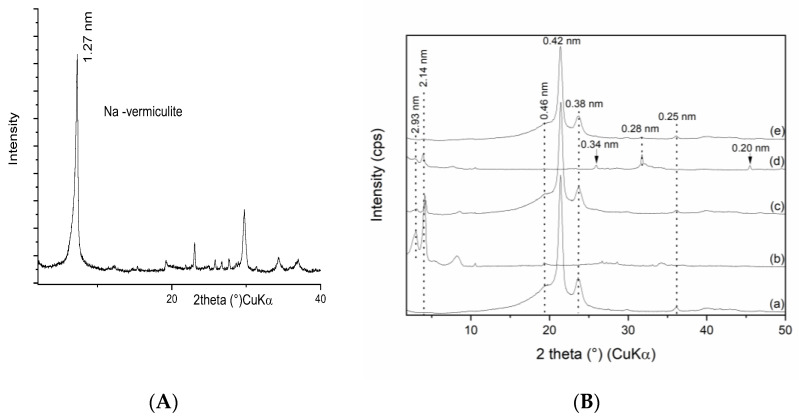
XRD patterns of the samples (**A**) monoionic Na-vermiculite, (**B**) (a) pure PE, (b) VCA, (c) PE/VCA, (d) VCAH and (e) PE/VCAH.

The XRD patterns of the pure Na—vermiculite (Figure 1A) and the set of PE composites and vermiculite fillers are shown in Figure 2B. The vermiculite intercalated with chlorhexidine diacetate showed two well-defined and intensive reflections at d = 2.933 nm and 2.14 nm [12] and residuum of the original non-intercalated peak. The PE/VCA showed a reflection of pure PE (d = 0.46 nm, 0.415 nm, and 0.376 nm) and intercalated vermiculite (d = 2.933 and 2.14 nm), where the intensity compared to the pattern in Figure 2B(b) was visibly lower. In this composite, the reflection of the PE remained without changes, and the intercalated vermiculite peaks positions were not changed; only the relative intensity of the V-peaks was lower (Figure 2B(c)).

The filler VCAH presented a reflection of the intercalated vermiculite (d = 2.933 nm and 2.14 nm) in accordance with [30,31] and reflections of the CDH (d = 0.343 nm, 0.282 nm, and 0.199 nm) (Figure 2B(d)). The intercalated vermiculite pattern peaks intensities were lower after modification with CDH.

Finally, the polymer composite PE/VCAH showed diffractions of pure PE and the reflection of CDH at 0.282 nm. The intercalated vermiculite was represented by a very low intensity peak at 2.14 nm (Figure 2B(e)). This pattern is expressing the acknowledged exfoliation of the vermiculite silicate layers; however, the decoration of the vermiculite with CDH remained evident.

The XRD pattern of the polymer composites confirmed the stable structure of the PE, the intercalation of the clay minerals with chlorhexidine diacetate, and the presence of CDH. The peak positions of all the components remained at the same values, indicating a lack of PE intercalation into the clay mineral interlayer or interaction with CDH.

### 3.2. Differential Scanning Calorimetry Structure Study

The crystallinity (Xc) of the polymer composites can be used to characterize the ratio of the crystalline part of the semi-crystalline polymer, and for the calculation of Xc, we used the following formula:

 Xc=ΔHm/ΔH100 *100%,

where  ΔHm is the enthalpy of melting during the heating process, ΔH100 is tabulated as the enthalpy of the crystallization–melting process. The ΔH100 of polyethylene is 293 J/g [32]. The crystallinity of the composites was lower than that of the pure PE. Fillers affect the crystallinity of the PE matrix shifting the values to lower numbers. A polymer composite with montmorillonite filler affects the polyethylene composite crystallinity less than vermiculite fillers. The crystallization temperature of the polyethylene composites compared to the pure PE decreased, but the melting temperature remained the same. (see Table 1).

### 3.3. Light Microscopy Imagining and Particle Size Distribution

**Figure 3:**
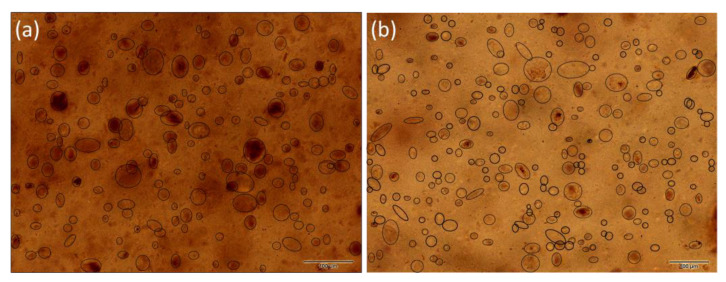
LM images of the samples with montmorillonite: (**a**) PE/MCA and (**b**) PE/MCAH with marked filler in the polymer matrix (scale bar 100 μm).

The distribution of the filler in PE matrix was studied using light microscopy imagining. Figure 3 shows the distribution of MCA in the PE matrix. The MCA particles in range 5–60 μm were dispersed in the PE matrix uniformly. The highest number of particles (44%) was in the range of 10–20 μm, the second most populated particles (32%) were in the range of 20–30 μm, the third were particles in the range of 30–40 μm at 17% (Figure 3).

In the case of the MCAH, the situation was different. The number of smaller particles was visibly higher—the amount was about 50% in the 10–20 μm range. The second in the range of 20–30 μm was higher than for the MCA at 21%. The third most common particles were in the 5–10 μm range. There were also particles in the range of 60–70 μm, which may be caused by the formation of large aggregates during the preparation of the polymer composite.

**Figure 4:**
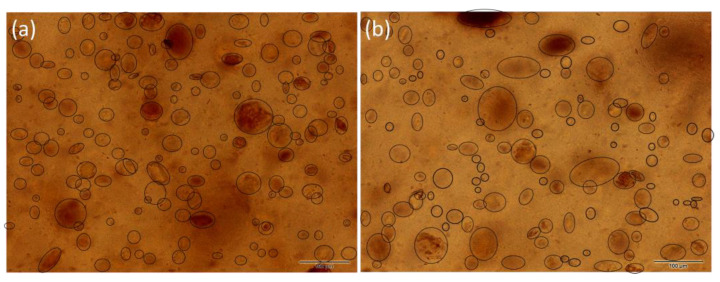
LM images of the samples with vermiculite: (**a**) PE/VCA and (**b**) PE/VCAH with marked filler in polymer matrix.

The PE/VCA showed a larger particle distribution than the PE with montmorillonite fillers (Figure 4). The particles were mostly in the 30–40 μm range (35%), the second interval was 20–30 μm (31%), and the third interval included particles in the 10–20 μm range (27%). The presence of larger particles may appear during the preparation of the composite.

The PE/VCAH composite showed the lowest number of particles compared to other polymer composites but contained larger particles that were likely to form aggregates. This composite showed clusters of particles around 100 μm (1% of particles) (Figure 4).

**Figure 5:**
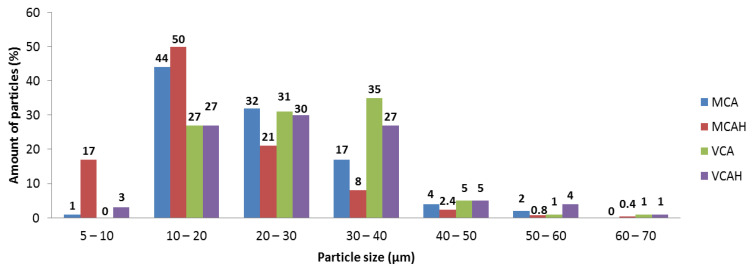
Histogram of the filler particles counted using light microscopy observation.

The distribution of fillers in all the polyethylene matrices was relatively homogeneous, which ensured the uniform presence of antibacterial and biocompatible parts on the entire surface of the studied polymer composites. In all the studied polymer composites, the particles were primarily in the range of 10–40 μm (Figure 5).

The particle size distribution (PSD) results of the fillers before compounding to PE are presented in Table 2. All the fillers median value was in a relatively narrow range from 11 to 15 μm. Generally, both vermiculite fillers were more uniform in size, and the median was higher. The montmorillonite fillers showed a trimodal or bimodal distribution. The wide distribution of MCAH and VCAH is due to the presence of the CDH that forms aggregates with clay minerals, as discussed in our previous study [33]. These findings are in correlation with the LM observation of the filler size in the matrix; however, the effect of the plate-like particles should be considered. Looking at the images acquired via transmission mode, we see the “flat particles” but not the one arranged perpendicularly, while the PSD method considers the particle as a 3D object. Therefore, the average particle evaluated by the LM was larger than the one from the PSD.

### 3.4. Antibacterial Test

**Figure 6:**
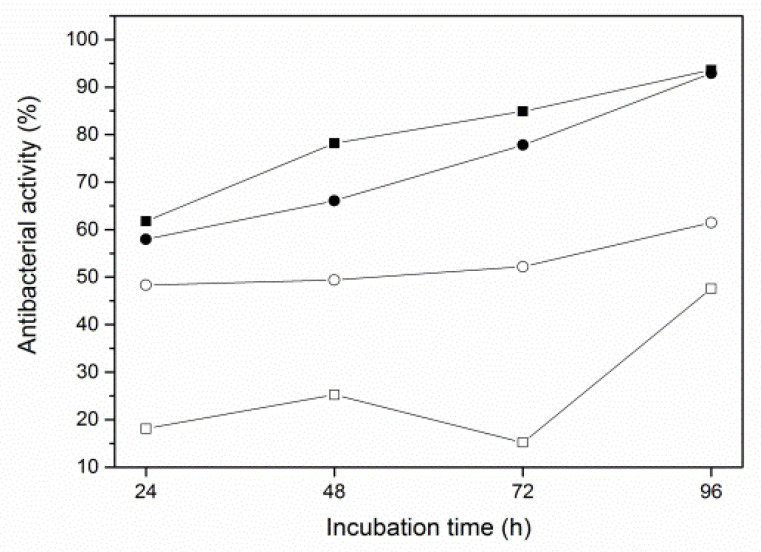
Antibacterial activity of the polyethylene composites: PE/VCA (▪), PE/MCA(●), PE/VCAH (□), PE/MCAH (○).

The antibacterial activity of the polymers containing clay mineral, hydroxyapatite, and chlorhexidine diacetate were compared with those containing only clay mineral and chlorhexidine. The surface of each sample was covered with a suspension of bacteria *Staphylococcus aureus*. The bacteria survived on the surface of clean PE plates throughout the experiment. The antibacterial activity of the MCAH was very low at the beginning of the experiment but increased rapidly after 96 h. In the case of the VCAH, the antibacterial effect was almost 50% and finally almost 60% (Figure 6, Table 3). This composite had very similar antibacterial activity throughout the test. The results in Figure 6 show that the composites without hydroxyapatite showed better antibacterial behavior, which may be due to the coating of the clay mineral surface with hydroxyapatite. The increase in the CA content in clay minerals should be investigated in the future.

### 3.5. In Vitro Biocompatibility

**Figure 7:**
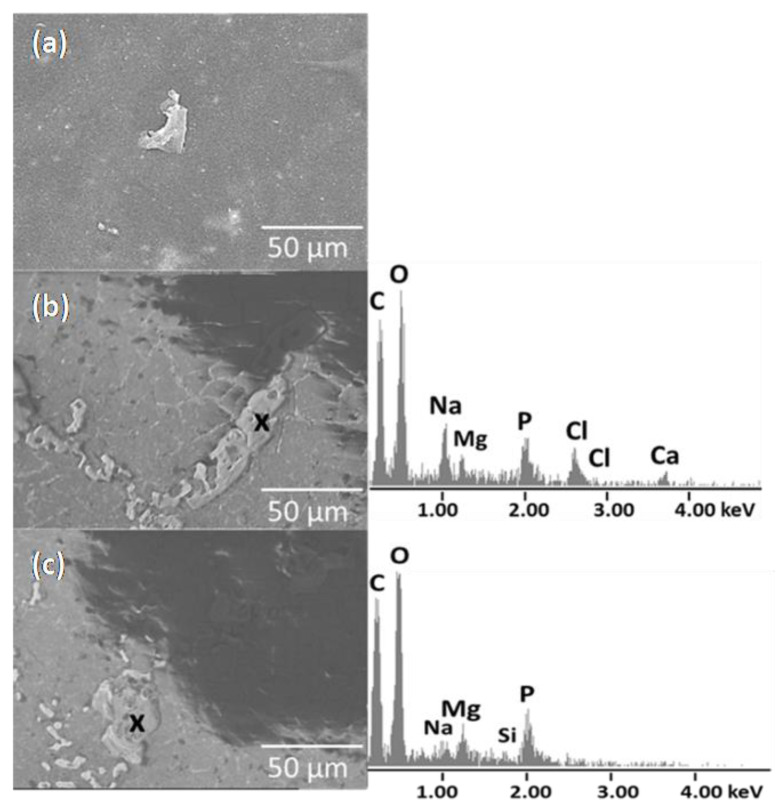
SEM images of the montmorillonite composites PE/MCAH before and (**a**–**c**) after immersion in SBF (elemental analysis EDS).

The surface of the PE/MCAH before immersion in the SBF was relatively smooth; in some cases, the filler particles probably protruded from the polyethylene (Figure 7). After incubation in SBF, the surface of PE/MCAH was covered with irregular different shapes with a bumpy surface. Elements corresponding to the PE matrix (C), the apatite phase (Ca, P, O), and other elements originating from the SBF liquid, such as Na, Cl, Mg, were detected by EDS elemental analysis. The surface irregularities and filler particles that appeared on the polymer surface helped to create an apathetic structure from SBF because the CDH contained in the fillers allowed ions in the SBF to bond with the Ca or P ions from the CDH [34].

**Figure 8:**
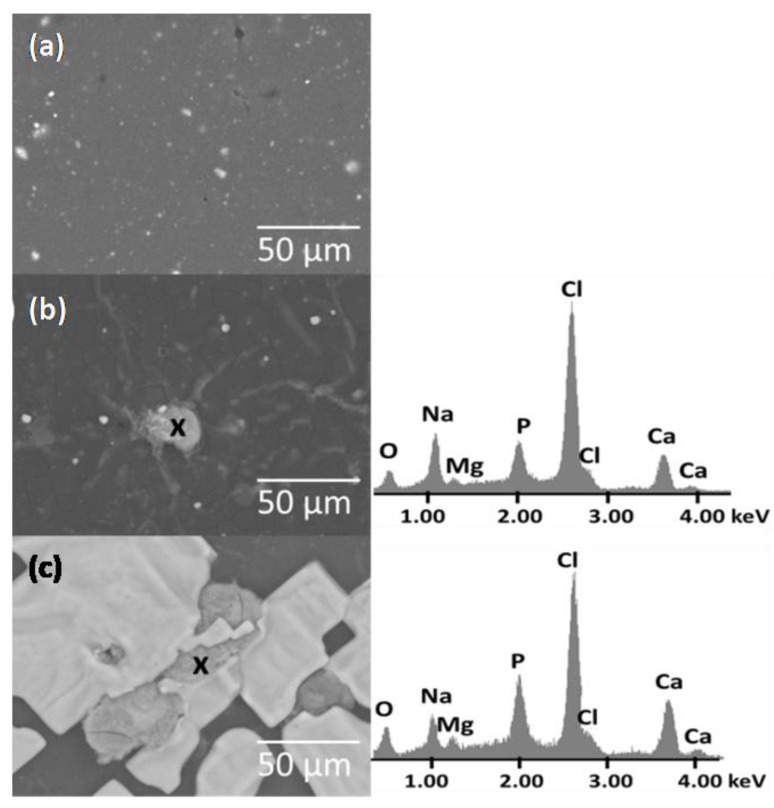
SEM images of the PE/VCAH before and (**a**–**c**) after immersion in SBF (elemental analysis EDS).

The surface of the PE/VCAH before and after immersion in SBF is shown in Figure 8. The surface of the PE/VCAH before immersion in SBF was relatively smooth with visible particles of fillers, which were not fully covered by the PE matrix. After immersion in the SBF, the surface was covered by a layer of small particles of apatite (Figure 8b) and with large crystals of NaCl or MgCl_2_ (Figure 8c). The EDS analysis confirmed elements from SBF, such as Na, Mg, Cl, and Ca.

## 4. Conclusions

In the study, the structure, the morphology, and antibacterial/biocompatible properties of the newly prepared polymer composite with hybrid organic–inorganic clay nanofiller were investigated. Polymer composites were investigated for their structural properties when clay nanofillers were intercalated with chlorhexidine, where intercalation was confirmed with an XRD peak shift. The next step of the CDH decoration of the intercalated silicate layers was confirmed through the independent peaks of the CDH phases d = 0.34, 0.28, and 0.2 nm. After mixing with PE, the clay nanofiller delaminated, and the reflection of the clays was not visible in the XRD pattern. Observing the clay particles’ distribution and homogenization in the PE matrix, we can state that montmorillonite dispersed better, creating smaller particles about 13 μm with more intensive exfoliation. The crystallinity of the composites during the temperature testing varied based on the filler type; vermiculite caused a lower crystallinity of the composite Xc = 33% compared to the one with montmorillonite filler Xc = 37%. Pure polyethylene was observed to show very poor antibacterial resistance, as the *Staphylococcus aureus* strain survived throughout the experiment on the tested surface. Nanoclay with chlorhexidine diacetate improved the antimicrobial properties and the subsequent modification of the clay with an inorganic biogenic compound (CDH) improved the biocompatible properties of the PE. The composite containing MCAH showed better antibacterial properties than the composite with VCAH; the biocompatibility properties looked very similar for both composites; so, the PE/MCAH appears to be the best candidate for further detailed study. Montmorillonite has the ability to intake a higher amount of intercalants to the structure; therefore, the antimicrobial effect can be increased by the amount of CA, and the dispersibility of the filler can be improved.

## Figures and Tables

**Table 1:** Crystallinity and the melting process parameters of the studied composites compared to the pure PE.

Sample	Tm/(°C)	Tc/(°C)	ΔHm/(J/g)	Xc/%
PE	108.83	93.4	111.9	38
PE/MCA	108.03	94.58	103.84	36.53
PE/MCAH	108.73	93.79	107.71	37.90
PE/VCA	108.74	93.78	89.95	31.65
PE/VCAH	108.44	93.82	101.27	35.63

Note: Tm—melting temperature, Tc—crystallization temperature, ΔHm—enthalpy of transition, Xc—crystallinity.

**Table 2:** Particle size distribution of the fillers MCA, MCAH, VCA, and VCAH.

Sample	Median (μm)	Mode (μm)	Distribution Type
MCA	11.10	12.31; 262.37	bimodal
MCAH	12.95	0.339; 12.35; 101.46	trimodal
VCA	15.75	12.41	monomodal
VCAH	13.51	12.30; 200	bimodal

**Table 3:** Average amount of CFU in three imprints.

Sample	24 h	48 h	72 h	96 h
PE/VCA	115	65	46	19
PE/VCAH	246	224	254	157
PE/MCA	126	102	67	21
PE/MCAH	155	152	143	116

## Data Availability

All data are mentioned in the manuscript.
